# Physical Tests for School Children

**Published:** 1921-02-05

**Authors:** 


### Physical Tests for School Children.

In an address to the members of the Manchester Literary and Philosophical Society, Dr. A. A. Mumford has been pointing out the necessity of having fresh physical-fitness tests for school children to replace those at present in use, because school work imposes a strain which increases as the child grows older. This is particularly evident in the indoor school life of the secondary-school child. It is perfectly true that, as dealt with in the medical inspection of schools, health has been mainly considered in its relation to exceptional children—the deformed, the diseased, the mentally unfit, etc. It has not been concerned with measuring the fitness of the child to put forth effort. Further tests of

**THE PUBLIC WELL-BEING**—(*continued*).

capacity to put forth effort are certainly needed, and these may be based either on the work of the heart, the lungs, or the nervous system

The new tests as described by Dr. Mumford are chiefly concerned with breathing. They have been brought into prominence, he says, by the work of the Air Force, and are now being adapted to boys in the Manchester Grammar School and to students in the Manchester University who volunteer for examination. The first test, dealing with the amount of air in the inspiration, is measured by the spirometer; the second, which deals with the force of the respiration, is measured by pressure against a column of mercury; the third relates to the movements of the chest, which are examined by means of a specially designed waistcoat. It would be interesting to know whether any valuable or definite results have been obtained from the experiments so far carried out.

